# Circ2Disease: a manually curated database of experimentally validated circRNAs in human disease

**DOI:** 10.1038/s41598-018-29360-3

**Published:** 2018-07-20

**Authors:** Dongxia Yao, Lei Zhang, Mengyue Zheng, Xiwei Sun, Yan Lu, Pengyuan Liu

**Affiliations:** 1 Sir Run Run Shaw Hospital and Institute of Translational Medicine, Zhejiang University School of Medicine, Hangzhou, Zhejiang, 310016 China; 2 Center for Uterine Cancer Diagnosis & Therapy Research of Zhejiang Province, Women’s Hospital and Institute of Translational Medicine, Zhejiang University School of Medicine, Hangzhou, Zhejiang, 310029 China

## Abstract

Circular RNAs (circRNAs), a new class of regulatory noncoding RNAs, play important roles in human diseases. While a growing number of circRNAs have been characterized with biological functions, it is necessary to integrate all the information to facilitate studies on circRNA functions and regulatory networks in human diseases. Circ2Disease database contains 273 manually curated associations between 237 circRNAs and 54 human diseases with strong experimental evidence from 120 studies. Each association includes circRNA name, disease name, expression pattern, experimental method, a brief functional description of the circRNA-disease relationship, and other detailed information. The experimentally validated miRNAs that may be ‘sponged up’ by these circRNAs and their validated targets were also integrated to form a comprehensive regulatory network. Circ2Disease provides a user-friendly interface to browse, search, analyze regulatory network and download data. With the rapidly increasing interest in circRNAs, Circ2Disease will significantly improve our understanding of circRNA deregulation in diseases and is a useful resource for studying posttranscriptional regulatory roles of circRNAs in human diseases.

## Introduction

Circular RNA (circRNA) is a type of RNA molecule which forms a covalently closed continuous loop by back-splicing or lariat^1^. Although circRNA was discovered decades ago, it was initially perceived as RNA splicing errors^2^. With the advent of next-generation sequencing (NGS), circRNAs were found to be abundant, stable and evolutionarily conserved among eukaryotes^3–5^. So far, tens of thousands of circRNAs have been predicted by bioinformatics methods. These circRNAs are largely classified into three categories, including exonic circRNAs^3^, circular intronic RNAs (ciRNAs)^6^, and retained-intron circRNAs or exon-intron circRNAs (EIciRNAs)^7^.

CircRNAs have become of great interest in the field of transcriptional regulation^8,9^. For instance, they can act as miRNA sponges^10^, or bind to RNA-associated proteins to form RNA-protein complexes that regulate gene transcription^11^. Some ciRNAs^6^ and EIciRNAs^11^ can interfere with pre-mRNA splicing that is a critical step in the posttranscriptional regulation of gene expression. Recent studies have identified circRNAs as novel regulators in many disorders such as Allzheimer’s disease^12^, diabetes mellitus^13,14^ and cancer progression^6^. Furthermore, due to lack of 3′ termini, circRNAs are more resistant to degradation by exonuclease RNase R and possess greater stability than linear RNAs^3,5,6^. circRNAs are abundant in blood samples^15^, saliva^16^, and exosomes^17^, making them promising diagnostic biomarkers for complex diseases such as cancer.

To facilitate studies on circRNAs, several circRNA databases have been developed, such as circBase^18^, Circ2Traits^19^, circRNADb^20^, CircNet^21^, deepBase^22^, CircInteractome^23^, TSCD^24^, CSCD^25^ and starBase^26^. Most of these databases integrated circRNAs information from high throughput sequencing studies and various datasets. For example, circBase combined data from several large-scale circRNA studies^18^. starBase displayed regulatory networks from thousands of circRNAs and CLIP-seq datasets^26^. circRNADb integrated datasets of circRNAs from literatures and their own datasets and contained 32,914 exonic circRNAs from various sources^20^. TSCD is a tissue-specific circRNA database from RNA sequencing (RNA-seq) datasets and characterized the features of circRNAs in human and mouse^24^. CSCD was focused on cancer-specific circular RNAs and identified more than 27 thousands of cancer-specific circRNAs^25^. Besides these, circNet also utilized transcriptome sequencing datasets to identify the expression of circRNAs^21^. Another database Circ2Traits studied the potential association of circRNAs with human traits and mapped trait related SNPs on circRNA loci^19^.

In addition to these large-scale studies, individual or specific circRNA deregulation in various diseases have been reported rapidly. However, these experimentally validated circRNA-disease associations are scattered among various studies. For this reason, we developed a high-quality circRNA-disease association database, named Circ2Disease. The current version of Circ2Disease contains 273 manually curated associations between 237 circRNAs and 54 human diseases with strong experimental evidence from 120 studies. The experimentally validated miRNAs that may be ‘sponged up’ by these circRNAs and their validated targets were also integrated to form a comprehensive regulatory network. With a user-friendly interface, users can easily browse, search, analyze regulatory network and download detailed information for each entry. We hope that this database can help in pushing forward the studies of circRNA-disease associations in the research community.

## Methods

### Collection of experimentally validated circRNAs

**Figure 1:**
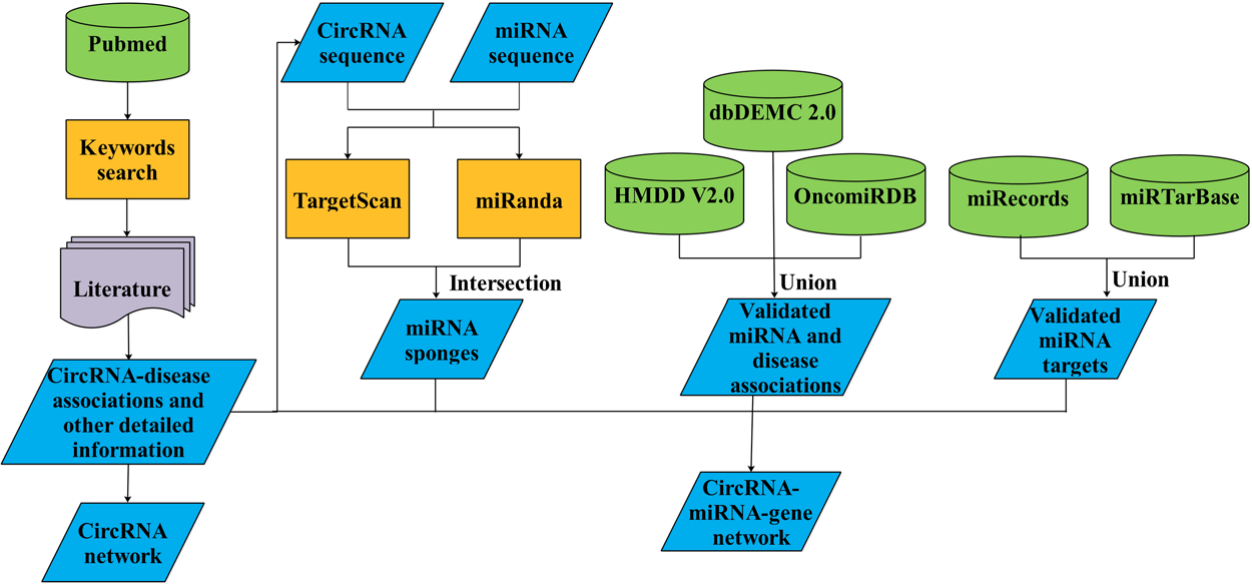
A flowchart of constructing Circ2Disease.

Figure 1 depicts the flowchart of constructing Circ2Disease database. We manually collected all the entries about the associations between circRNA and human disease. Firstly, we searched the PubMed with a list of keywords, such as ‘circular RNA disease’, ‘circRNA disease’, ‘circular RNA cancer’, ‘circRNA cancer’, etc. All the abstracts were viewed to determine whether a study is associated with circRNA deregulation and human disease. Secondly, we carefully reviewed all related literature published prior to 1 November 2017 and curated the detailed information of circRNAs with experimental evidence. To provide high-quality associations with strong experimental evidence, circRNAs only identified by microarray or RNA-seq were not included. If any miRNAs that are sponged by circRNAs were validated experimentally, their miRNA targets, RNA binding protein (RBP) and other up- or down-stream regulatory genes were also retrieved from literature. Thirdly, circRNA and disease names were normalized. In literature, the names of circRNAs and diseases varied from one to another study. To be more readable, we normalized their names in a uniform way. For circRNA names, we unified the names on the basis of circBase, Human circRNA microarray (Arraystar, USA), their original names in particular papers, etc. The names representing the same circRNA were grouped as a collection. Each collection was assigned a name according to the order: original name, circBase id, Arraystar probe id, and others. All these groups are listed on: http://bioinformatics.zju.edu.cn/Circ2Disease/circRNAgroup.html. The disease names were also normalized based on the standardized classification vocabulary of Disease Ontology (http://www.disease-ontology.org/).

### CircRNA-miRNA-gene network construction

As circRNAs may act as miRNA sponges, we predicted potential miRNA response elements on these circRNAs using software TargetScan^27^ and miRanda^28^. We separately ran TargetScan with default parameters and miRanda with “strict” parameters. To increase the specificity, 6mer matches were excluded from the TargetScan result. The intersection of the two results was considered as the predicted miRNA sponge. To determine the interactions between these miRNAs and diseases, we integrated three public databases, i.e. HMDD V2.0^29^, dbDEMC 2.0^30^ and OncomiRDB^31^. Experimentally validated miRNA targets from miRecords^32^ and miRTarBase^33^ were also included in the circRNA-miRNA-gene network. For data from miRTarBase, only strong experimental evidence of miRNA–target interactions (MTIs) were retained for further network analysis. The RNA-binding proteins (RBPs) potentially interacted with circRNAs were retrieved from CircInteractome database^23^. All these interactions formed a comprehensive circRNA-miRNA-gene regulatory network, which will help users elucidate the pathogenesis of diseases and offer beneficial evidence for diagnosis and therapy.

## Results

### Browse and Search

**Figure 2:**
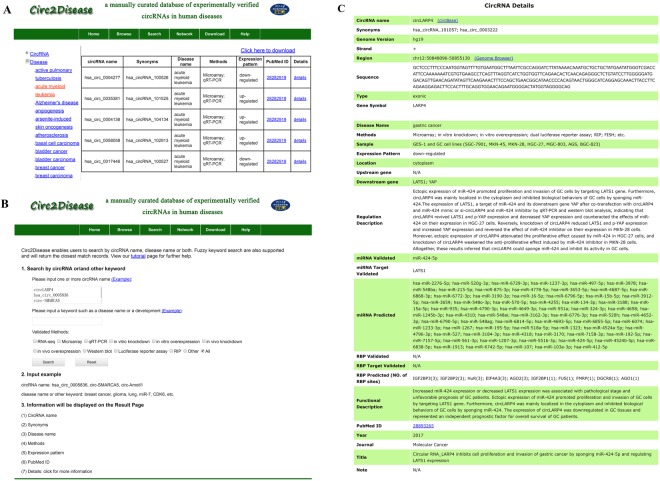
Detail information on the Circ2Disease webpage. (**A**) Browse function. (**B**) Search function. (**C**) Detailed information of circRNA.

A user-friendly interface was provided in Circ2Disease to facilitate users to browse, search, analyze regulatory network and download all the detailed information on circRNA-disease associations. In the ‘Browse’ page, a specific circRNA or disease name, and a list of matched entries can be browsed by clicking and a list of matched entries will be displayed on the screen (Fig. 2A). In the ‘Search’ page, Circ2Disease allows users to query detailed information on each circRNA-disease association by circRNA name and alias name, and/or disease name (Fig. 2B). Circ2Disease also offers fuzzy keyword search function, allowing users to retrieve the closest matching records. In addition, users can filter associations by certain experimental methods. Each item in the result provides a link that takes the user to the detailed circRNA page (Fig. 2C).

### Network analysis

**Figure 3:**
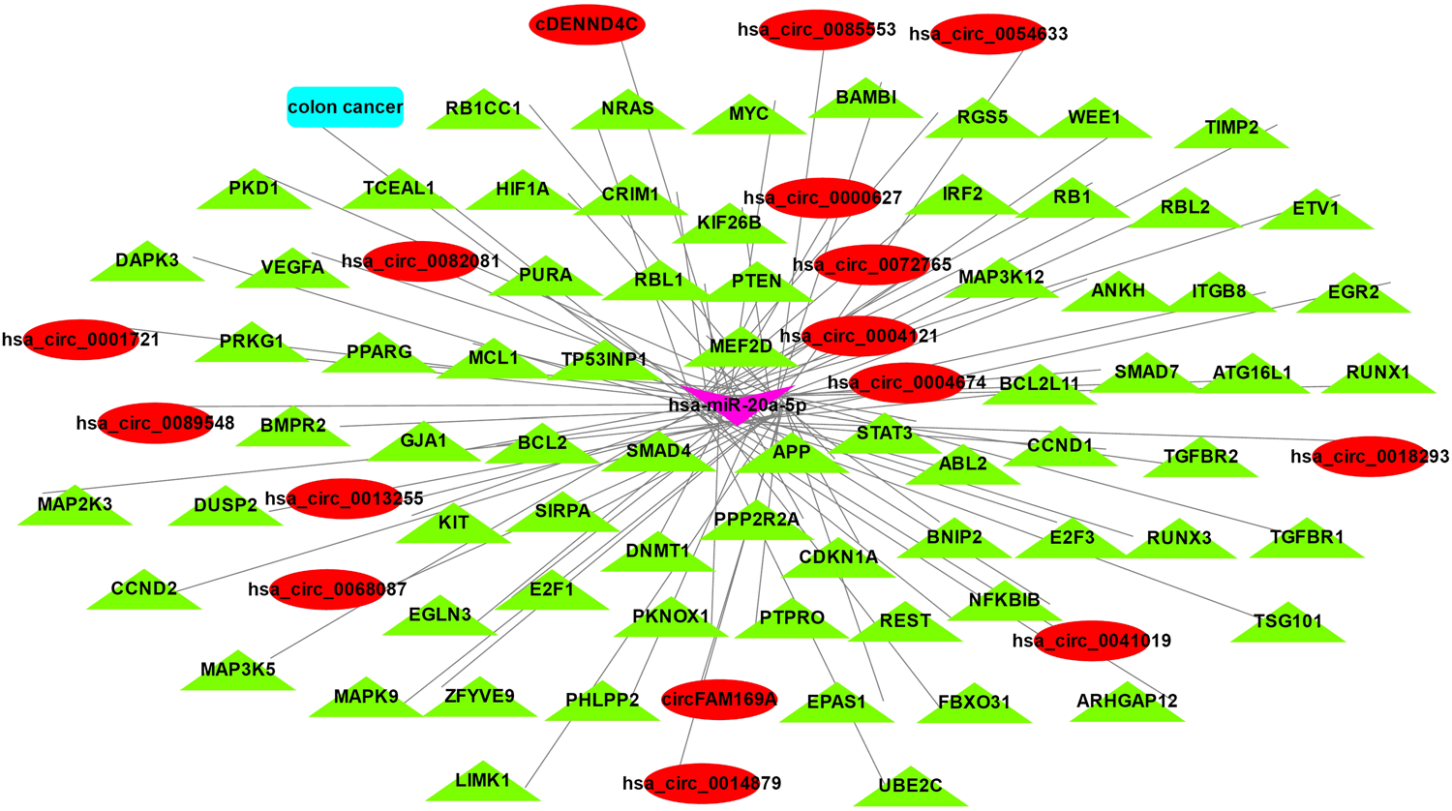
hsa-miR-20a-5p search results in CircRNA-miRNA-Gene network.

Two pages were provided for network analysis in Circ2Disease. They can be accessed by hovering the mouse on the ‘Network’ menu. By clicking the ‘CircRNA’ page, a network of all the circRNAs and their up- and down-stream genes that were manually retrieved from literature will be displayed. ‘CircRNA-miRNA-Gene’ page allows users to search and tune all regulatory networks across circRNA studies (Fig. 3). Users can input circRNA name, disease name, miRNA or gene to search the network. After submitting the query, all the matched entries will be organized as a visualizing network. There is a toolbar above the network. Using the toolbar, several functions can be executed:

1. Search: circRNA, miRNA, disease and gene can be input to search the network and is case-insensitive. The matched node will be turned yellow and displayed in the center of the page.
2. Zoom: Scroll the mouse wheel to zoom in and out of the network.
3. Display: Grab and drag nodes; tap a node or edge to select; tap blank to unselect.
4. Hide/Show: The selected nodes and edges will be hidden or shown.
5. Neighbor: Click the “Neighbor” button to select nodes and edges connected to the previously selected nodes.
6. Export: Export the displayed network as JPG or PNG file. It can be saved on a local computer.

All these graphical networks were developed based on the cytoscape.js^34^. We used different colors and shapes to represent the nodes and edges: red ellipse nodes indicate circRNAs, cyan roundrectangle nodes indicate disease names, purple vee nodes indicate miRNAs and green triangle nodes indicate genes.

### Download and Help

All the entries can be downloaded in the ‘Download’ page, and a detailed tutorial of Circ2Disease is available on the ‘Help’ drop-down menu. A more detailed user guide can be found on the tutorial page.

### Case study 1: circRNA dysregulated in breast carcinoma

To identify circRNAs which are potentially involved in breast carcinoma, we searched a keyword “breast carcinoma” in Circ2Disease. As a result, 29 circRNAs were found in Circ2Disease. Among them, seven circRNAs had been previously assigned a function by direct experimentation. They are circ-ABCB10, cDENND4C, circ-Amotl1, circ-Amotl1, hsa_circ_0006528, circ-Foxo3 and circGFRA1. The functions of the other 22 circRNAs remain unknown. In the present study, our analysis was focused on these novel downregulated circRNAs, including hsa_circ_0000911, hsa_circ_0018293, hsa_circ_0001283, hsa_circ_0000893, hsa_circ_0000981, hsa_circ_0006054, hsa_circ_0004619 and hsa_circRNA_406697. In addition, Circ2Disease provides RBP-circRNA interaction results, which predicted that hsa_circ_0001283 can bind to AGO2. This indicates that hsa_circ_0001283 may act as a miRNA sponge.

BRCA1 is a tumor suppressor gene in human^35^; its protein, also called breast cancer type 1 susceptibility protein, is responsible for repairing DNA in breast carcinoma^36^. According to miRTarBase, BRCA1 is an experimentally validated target of 27 miRNAs such as hsa-miR-146a-5p and hsa-miR-15a-5p. Interestingly, hsa-miR-146a-5p is one of miRNAs that were predicted to target hsa_circ_0001283 in Circ2Disease. Taken together, these results suggest that hsa_circ_0001283 can bind to hsa-miR-146a-5p to regulate the expression of BRCA1 in breast carcinoma. By combining and mining from the validated or predicted circRNA, RBP, and other information such as miRNA-mRNA target interactions, we can prioritize the circRNA-disease association and generate testable hypothesis for further investigation.

### Case study 2: circRNA-miRNA-gene network

The miRNA set analysis using TAM tool^37^ demonstrated that hsa-miR-20a-5p was significantly enriched in immune system, HIV latency, cell proliferation, angiogenesis and apoptosis. This makes it as an attractive candidate potentially involved in cell signaling and disease^1^. Thus, we searched hsa-miR-20a-5p in circRNA-miRNA-gene network in Circ2Disease. The network analysis found 71 hsa-miR-20a-5p validated targets such as CDKN1A, VEGFA, and MYC, and 16 circRNAs that were predicted to bind to hsa-miR-20a-5p (Fig. 3). Then, we discovered that these 16 circRNAs were dysregulated in multiple diseases (e.g., cornonary artery disease, type 2 diabetes mellitus, osteoarthritis, gastric cancer and so on) by keyword search in “Search” page. Together, these results indicated that these 16 circRNAs may act as sponges to hsa-miR-20a-5p to regulate gene expression of the 71 targets in different diseases. These hypotheses generated from Circ2Disease are worth being investigated in the future.

## Discussion

Circ2Disease is the first database for experimentally validated disease-related circRNAs. These data provide the missing cornerstone of functional studies of circRNAs in human diseases. Circ2Disease also constructed a comprehensive circRNA-miRNA-gene network and gives deeper insights into the posttranscriptional regulatory roles of circRNAs in diseases. As the number of circRNA-disease association increases in the future, we will continue to integrate those data into Circ2Disease and will update the database regularly. More importantly, as only a small number of circRNAs have been characterized to be associated with diseases, it is necessary to incorporate more data sources and bioinformatics methods to improve the utility of this database. We believe that Circ2Disease would be a valuable resource for studying circRNAs and will significantly improve our understanding on the relationships between circRNAs and diseases.
